# The Nursing Supplement

**Published:** 1888-07-14

**Authors:** 


### The Nursing Mirror.

All Contributions for this Supplement should be addressed "The Nursing Mirror," care of The Editor, 38, Old Jewry, E.C.

### En Passant.

STAFFORDSHIRE INSTITUTION FOR NURSES.—In our notice of this institution on June 16th, we stated that £10 had been levied in fines from nurses during the past year. The matron writes to explain that we had mistaken the statement made in the balance-sheet, and that this £10 was a half-year's salary forfeited by a nurse who had broken her agreement to remain three years on the staff. We are very glad to publish this explanation of a ludicrous mistake, for the maintenance of discipline by the exaction of fines always shows a wrong spirit in the management of an institution. There are actually some convalescent homes where the patients are fined for spilling the ink, &c.; we cannot help thinking that the word "home" is misapplied to such places.

THE WOMEN'S JUBILEE OFFERING.—There has been a correspondence in the *St. James's Gazette* on the subject of St. Katherine's Hospital, one writer maintaining that it was not the sort of charity with which the loyal gift of the women of the empire should be connected. In our last week's issue we gave an account of the hospital, which has certainly in the olden days often suffered from the rapacious habits of its officials. All the more need it should now be put on another footing. Mr. Rathbone, M.P., who founded the Liverpool Nursing Institute, has joined the committee of the fund, to give it the aid of his practical experience. All will be under the sole control of the Queen. The surplus of the offering is to purchase a necklace of pearls and diamonds for her Majesty, made to represent a wreath of trefoils, after a design by Princess Beatrice.

MRS. ERNEST HART.—This noble worker in the cause of the poor of Donegal has been the recipient of much sympathy and congratulation since she has so thoroughly vindicated her labours before all the world. At a crowded meeting held in the theatre of the Royal Dublin Society last month the following resolution was proposed and carried by acclamation: "That this meeting entertains a strong sense of the public value of the work carried on by Mrs. Ernest Hart in Ireland and of the technical teaching in connection with the Donegal Industrial Fund described in her address, and it tenders to her an expression of sympathy on her patriotic labours, and of admiration for the devotion and ability with which she has carried them out." There were present at the meeting Sir Roland Blennerhassett, the Rev. Dr. Molloy (president of the Catholic University), Mr. Commissioner Lynch, Mr. Fahie, and others. Mrs. Hart delivered an address explaining her method, and exhibited some of the work done, such as is shown at 43, Wigmore Street. In our Annotation of June 16th we mentioned that the sum of £5,000 is needed to make Mrs. Hart's work a complete success, and we hope that some of our readers will show their sympathy with this lady during her late troubles by subscribing towards this sum.

SCHOOL OF MEDICINE FOR WOMEN.—Miss Louisa Stevenson presided last week at the opening meeting of the London School of Medicine for Women, held at 30, Henrietta Street, Brunswick Square. The school is in connection with the Royal Free Hospital in Gray's Inn Road. The report of the school, read by Mrs. Garrett-Anderson, stated that at the beginning of the winter session 1887-88, 19 students entered for their first year, and took two single classes. There were now 77 students attending the school, and of these 38 were receiving clinical instruction at the Royal Free Hospital. In conclusion, the council thanked their kind supporters for their assistance in maintaining the school, and trusted that their endeavours to provide women with full facilities for the study of all branches of medical science as far as was possible in a small school might continue to be satisfactory. Miss Stevenson, who has lately visited India, then gave an account of her impressions of the work of medical women in that country, pointing out some of the difficulties, especially those of a monetary character, and addressed a few words of encouragement to the students. She afterwards distributed the prizes and certificates, and the proceedings closed with a vote of thanks to Miss Stevenson, moved by Mr. Ford North, seconded by Surgeon-Major McNaley.

THE NEW HOSPITAL FOR WOMEN.—Under the presidency of the Lord Mayor, a meeting was held on July 6th in aid of the building fund of the New Hospital for Women in Marylebone Road. In 1866, St. Mary's Dispensary for Women and Children was opened in a poor district of the parish, to afford women the opportunity of obtaining advice from duly-qualified medical women. The dispensary became a hospital in 1872, and by the liberality of friends has been enabled for the past sixteen years to carry on its good work at 222 and 224, Marylebone Road. By the expiration of the lease the management have been forced to contemplate the necessity of building new premises. Negotiations for the acquisition of a suitable site close to St. Pancras Church, in the Euston Road, have been entered upon. Hospital buildings will cost £10,000. The committee therefore desire to raise £20,000 to enable them to secure the freehold. Towards this sum close upon £8,000 has been obtained. Last Friday's meeting was held for the purpose of appealing for the residue. The total current expenses for twenty-six beds, including rates and taxes, salaries, wages, and repairs, and the maintenance of the in-patients, as well as all the expenses of a very large out-patient department, have been about £2,000 each year. There is no debt upon the current expenditure of the hospital, and the Committee believe they could continue to meet the expenses in the larger building if relieved of the burden of rent. The experience gained at the hospital is of the greatest advantage to the medical women on the staff, and to the students who attend the hospital. Young medical women who are intending to go to India, China, or Africa, obtain at the New Hospital training in the treatment of the diseases of women which at present they can get nowhere else. The demand for medical women in India is constantly increasing. Lady Dufferin's committee have repeatedly asked for more women for India than have been found willing to go; and one reason has no doubt been that the junior practitioners have in some cases felt that they have not had the amount of independent practice and experience which the responsible work in India requires. It must be remembered that there is a large step between even the most diligent student, who as such does nothing independently, and a hospital doctor, who must be prepared to undertake every sort of case, surgical and medical, without the aid of skilled assistants, or even of trained nurses. It might often be the duty of a medical woman in India to perform a serious operation which she had only seen done perhaps once or twice in her student time, and which no man would think of performing unless he had received training as an assistant to a surgeon, or as a house-surgeon in a hospital. The Royal Free Hospital, invaluable as is its practice to the female students, does not at present admit them to the resident posts, nor does it devote any large number of beds to the treatment of the special diseases of women. A letter from Miss Nightingale was read, wherein she expressed her sympathy with the hospital as a training college for medical women.

### District Nursing.

District Nursing has two distinct branches: that which prevails in the metropolis and large towns, like Leicester and Manchester, and that which prevails in country villages and the smaller towns. Nearly all the district nursing done in London is founded on the scheme of the Metropolitan and National Nursing Association, which has been working more than ten years, and has started branch homes at Holloway, Paddington, Clapham, Hampstead, Kensington, and other places, the central offices and training school being at 23, Bloomsbury Square. A candidate who wishes to receive thorough training in district work has, on entering the Central Home, to state in writing her intention to continue district nursing for a period of two years after her course is complete. A month's trial is given to every candidate, during which time she goes about with the nurses, and has her eyes fully opened as to the duties she is undertaking. If still stedfast in her resolution she is passed on to St. Thomas's Hospital for a year's experience of hospital work, and after that she has another three months or more at the Central Home, during which she must attend lectures and pass a final examination. The fees for this course of training include £5 for the first trial month, £30 for the year in hospital, and £3 a month during such further training as is necessary. For these fees a probationer has full board, including extras, an allowance for washing, uniform dress, and a separate furnished bedroom. Sometimes a probationer may get these fees remitted, but such chances are rare. The nurses on the staff of the Association receive from £30 to £50 a-year, while even higher salaries are secured for some who are sent to various posts. The rules under which the nurses work are strict, and their duties are often arduous. The breakfast-bell rings at half-past seven, and by eight o'clock the nurses, in their bonnets and cloaks, are ready to start on their rounds. Once a-week each nurse is accompanied by the lady superintendent, who keeps a sharp look-out for untidy bandages or dusty corners, and is ready to listen to the complaints of the patients. Nurses are responsible for the personal cleanliness of each patient under their charge, and also for the state of the room. If there is no relation or friend to do the rough work, the nurse must tuck up her sleeves and scrub the floor and black the grate. This, however, seldom happens, and the usual duties consist in washing the patient, doing the hair, making the bed, washing all crockery and utensils, and attending to the doctor's orders with regard to outward applications or the giving of medicines. At a quarter to one the nurses return to the home for dinner, after which they have two hours for rest or recreation. Tea is served at four o'clock, and then the nurses sally forth once more on their rounds till the supper hour, eight o'clock, arrives. Of course many cases have to be visited twice a-day or oftener; and occasionally a nurse has to sit up at night or take charge of a fever case, when special arrangements have to be made. There seems to be only one fault about the Metropolitan and National Nursing Association, and that is that they will have nothing at all to do with confinement cases. Seeing how large a number of women in need would be the better for thorough nursing at such a time, it appears a pity that the Association cannot have at least one trained midwife at each of its centres. No class is more ignorant, superstitious, and given to drink than the midwives and monthly nurses who attend the poor in our large towns. Only a short time since we heard a patient say of such a woman that she never touched a drop of beer, but always took six or seven glasses of brandy a-day to keep her up. She used also to carry about a bag in which she had various instruments of which she was very proud, and whether it was necessary to use them or not she always displayed them by the patient's bed. This woman had only one good quality, that her power of talking and lordly manner inspired her patients with belief in her power to do.

The other branch of district nursing is that prevalent in our country towns, where one nurse does the whole work, or as much as she can. In these cases the nurse generally works under a committee consisting of the clergyman and district visitors of the place, and so far as rules go her conscience is her only law. She probably secures lodgings in some respectable family in the poorer part of the town, and her salary seldom exceeds £70, out of which she has to meet all expenses. The life is very lonely in so far as her work is concerned, for there will be none but the parish doctor to give her a hint on nursing, or a word of encouragement when a difficult dressing is well done. Nurses living alone like this are very apt to undertake too much, to be careless about their food, irregular about their hours of sleep, and completely forgetful of the necessity of recreation. The old methodical habits learnt at the training-home are gradually abandoned, and the nurse does not see that though she works harder she does not get through so many duties as when under strict rule. The patients are more respectable than those nursed in larger towns, and the idea of a nurse who is a lady sweeping their rooms for them and making their beds is regarded as too strange not to be protested against. They are used to the district visitor, who brings them jelly and reads to them, and unless the nurse is careful they will relegate her to the same sphere. They will promise to make the bed thoroughly, and declare they can move the patient easily, and even promise to use the carbolic soap. But all these things can only be done properly and pleasantly by the nurse, who, as soon as she sinks to the mere dispenser of charity, has put at nought all her years of training and sunk to the level of an unprofessional woman. This is only a word of warning to those who seek country work. It is not to be supposed that all nurses behave thus, still there is the temptation to become lax as a natural rebound from the discipline of the training school, and nurses in small towns must guard against it. On the whole, the work in London, where all is carefully organised, and the path of duty is plain before one, is far easier than trying to please an uncomprehending committee, who probably only worry you in their desire to help. The dream of greater liberty which lures many a nurse into seeking a situation unconnected with an institution, often proves a delusive vision. To sit up till twelve loses its charm when there are no fellow-nurses to chat with; to lie in bed till ten is no treat when, whatever the hour, you have your own breakfast to cook. To work under a superintendent whose keen eye is trained to discover faulty nursing is easier than to have half-a-dozen untrained people always complaining of what are not faults, so that it is not in search of easier work that a nurse should seek an isolated situation.

There is one other small branch of district nursing, and that is when a nurse is attached to a dispensary, and attends such patients as require it at their own home. It is not a very satisfactory way of working, though there is plenty to be done, and the cases are generally varied. The excitement of hospital life is, of course, found in no branch of district work, where surgical cases are few and far between. Chronic, incurable, and bedridden patients are always in the majority on a district nurse's register, whether she works for an institution, a dispensary, or a committee.

---

"The neurotic woman"—that is, put in common language, the nervous woman—"is sensitive, zealous, managing, self-forgetful, wearing herself for others; the hysteric, whether languid or impulsive, is purposeless, introspective, and selfish. In the one is defect of endurance, but in the other defect of the higher gifts and dominion of mind."—*Clifford Allbutt, M.D.*

### Anecdotes of Personal Adventure.

### A MAUVAIS QUART D'HEURE.

It is many years since I had the following adventure, but it made such a vivid impression on my mind that the most trivial detail of the scene is indelibly imprinted on the "tablets of my memory." I was then night superintendent of a large lunatic asylum. One afternoon the patients had been out for their usual constitutional, when on returning through the airing grounds one of the women contrived, unobserved by either nurses or patients, to hide behind one of the bushes until the rest were indoors. She then darted up a ladder, which had been carelessly left by some workmen who had been repairing the roof, and so reached the top of the building, which was three storeys high. I was coming downstairs to tea at the time, and saw through a window in what a perilous position the woman was, for I recognised her as being one of our most determined suicidal cases. On the impulse of the moment I rushed up the ladder after her. By this time she was standing amongst a nest of chimney-pots with her back turned to me, and did not know that anyone was after her. She was just going to take a sensational "header" into the court below, when, to her intense surprise and disgust, I flung my arms round her, thus fastening hers to her sides, lifted her up in the air, and then threw her down between the two rows of chimneys. I seated myself on her prostrate form, and held on to her for dear life, for well I knew the dread alternative if my nerve or physical strength gave way. She implored me to let her go and end her miserable life, and threatened to throw us both down if I would not. "But you cannot, my good woman," I said, "I am just as determined as you are, and a great deal stronger." And so she found, for in spite of her kicking, and scratching, and biting I was able to keep my position until the arrival of the workmen to our aid. I was told I was perched there a good ten minutes. It seemed like as many hours. The men tied ropes round the woman's waist, knees, and under her arms, and then let her slide down the ladder to the anxious group gathered below. I suppose I came down to terra firma in a similar manner, but "the subsequent proceedings interested me no more," as Bret Harte puts it, for I had fainted. It was a long time before I was my usual self again. I used to go over the whole thing with any number of variations in my dreams and wake in a fright.

Our medical superintendent first gave me a good scolding for risking my life on such a forlorn hope, and in the next breath said I deserved the Victoria Cross. I suppose it was rather a foolish thing to do, but at the time I merely considered that

"I did my duty and never thought
I did any more than a woman ought."

A. E. B.

### "ALL'S WELL THAT ENDS WELL."

I was once sent by the Training Institution to which I then belonged to nurse a case of small-pox. At that time there was no fever hospital in the town as there is now. In order to isolate us as much as possible, a wash-house at the top of the yard was converted into a temporary sick-room; our food, &c., was set down at a respectful distance for me to fetch. I had to get what sleep I could on a shake-down, for my patient was by no means wealthy; and besides, there was no accommodation for another nurse. We got on fairly well for about a week, until one terrible night my patient was very delirious and would persist in trying to go outside. So I had to lock the door, pocket the key, sit by his side, and do my level best to keep him in bed. This sort of thing had been going on for some hours, and I had just decided that "I would not spend another such a night, though 'twere to buy a world of happy days," and that I must have more help, when my patient sprang out of bed. To knock me down, leap on to the sink, unfasten the window which opened outwardly, jump through, and so into the street, took Mr. —— less time to do than it takes me to tell it. He was simply and airily clad in his nightshirt, and it was about four o'clock on a dark winter's morn and was snowing heavily. Of course I went off in pursuit, but could find no trace of him. I returned, roused his friends, asked them to give information to the police, and went back to my wretched hovel, where I spent a very "bad quarter of an-hour." Naturally I thought that when I next saw him he would be dead or dying. Completely broken down, I was indulging in the feminine luxury of a good cry, when the lost sheep returned to the fold. He took no notice of poor me, but sat down by the fire. I soon got him inside a dry shirt and outside some hot beef tea, and into bed. He slept for hours, and seemed no worse for his impromptu snow-bath. The doctor said it had done him good; still I never heard that he ever prescribed this heroic course of treatment. We fully expected to hear that a ghost had been seen in the vicinity, but we never did. I suppose no one was out that wild, inclement morning but my patient, myself, and a stray policeman or so, and we know they do not see everything. So we never knew where Mr. —— went for his constitutional, nor how he managed to find his way back by himself in the state of mind he was in at the time.

A. E. B.

### ST. MARYLEBONE INFIRMARY, NOTTING HILL.

The fourth anniversary of the opening of the training school for nurses in connexion with this infirmary, by her Royal Highness the Princess Christian, was held in the home on the 25th June. The probationers who are about to finish three years' training having been subjected to a written and *viva voce* examination by Mr. Lunn, F.R.C.S.Ed., the medical superintendent of the infirmary, and Mr. Croft, F.R.C.S., of St. Thomas's Hospital, Mr. Boulnois, J.P., Chairman of the Infirmary Visiting Committee, and Mr. Bonham Carter, Secretary of the Nightingale Fund, addressed the meeting. During the year nine probationers were taken on the staff of the infirmary as nurses. Of former probationers, five have received the following appointments: Head nurse to the St. Marylebone Infirmary; head nurse to the Darlington Hospital and Dispensary; nurse to the Home for District Nurses, at 510, Edgware Road; nurse to the City Poor Law Infirmary, Edinburgh; and head nurse to the Paddington Infirmary, Harrow Road. The guardians of St. Marylebone parish, in conjunction with the committee of the Nightingale Fund, carry on this much-needed work of providing nurses for the sick poor; they have bestowed much thought and care on all their arrangements, and may be congratulated on the success which has attended their efforts. The home is built on the most approved plan; it is liberally supplied with baths, and everything conducive to the health of its occupants, and each probationer has a separate bed-room. Lectures and classes are carried on in the large class-room of the home, and are well attended. Probationers are bound to the Infirmary Committee for three years. Having passed through their year of training, those who are drafted on to the infirmary staff receive a salary commencing at £20 and rising to £25 per annum, with uniform and washing. Having finished their first and second year of service satisfactorily, they receive in addition, from the Committee of the Nightingale Fund, a gratuity of £2 for their first, and also for their second year of service. A letter of approval accompanies each gratuity. Many nurses are now in receipt of these tokens of good work. Papers containing the regulations for admission to the school may be obtained of the matron, Miss Vincent.

### Appointments.

[It is requested that successful candidates will send a copy of their applications and testimonials, with date of election, to The Editor, The Lodge, Porchester Square, W.]

The Queen's Hospital, Birmingham.—Miss M. L. Roberts has been appointed lady superintendent of nursing at the Queen's Hospital, in place of Miss Fletcher, who is appointed matron of the Royal London Ophthalmic. Miss Roberts is the daughter of a clergyman, and for some years before she entered the nursing profession she had the care of district work. In 1882 Miss Roberts became a probationer at St. George's Hospital, and in 1884 was made a sister, which post she has held for four years. It is with great regret that many friends will part with Miss Roberts in August, but still all feel she cannot refuse a wider scope for her capabilities.

Broomhill Home, Kirkintilloch.—At a meeting of the directors of the Association for the Relief of incurables for Glasgow and the West of Scotland, held on July 6th, Miss Phœbe Winder, late of Adelaide Hospital, Dublin, was unanimously elected matron of the Broomhill Home, though there were nearly sixty applicants for the post.

Ipswich Fever Hospital.—Miss Keer of Ipswich has been appointed matron of the Infectious Hospital in that town.

### Everybody's Opinion.

[*Correspondence on all subjects is welcome, but correspondents would oblige by writing on one side of the paper only.*]

### PATIENTS AND VISITORS.

Very interesting and very desirable is your news of hospitals and nurses; but may I be heard if I speak specially of their patients—the patients it is my lot to visit in the Hope Infirmary—union patients, too often ranked low in the social scale, even of gratitude, and most mistakenly so, in my experience. Again and again I hear their well-deserved praise of the doctors and nurses. "We couldn't be better done by if we paid ever so much," they say; and their belief in their perfect treatment is constant and assured. Nor is their gratitude confined to the staff—and this I must dwell upon, as I desire to induce more hospital visiting, to cheer a little the monotony that must be in a union infirmary, however pleasantly the walls are decorated and the floors polished, however well chosen the books, however much considered the daily comfort of the beds and lockers—those treasures of individual possession that make a home of the few feet of space that can be assigned to personal occupation in a hospital ward; the outside voice is needed to refresh their minds and enlarge them with the world's interests, and that better view of things that is never far to seek in the smallest events. Nowhere so much as in a hospital, where the worry of ways and means and the general combat of life are laid aside, are minds and hearts so truly the "blank paper" to receive impressions, to welcome words of faith, of courage, of friendship, of recommendation, not forgetting the admonition to show kindness by word or deed, or look even, which possiblility of doing is a help and happiness to many a patient who feels the night come—too soon, perhaps, when there is no more work; and great is the influence of such kindliness, and indeed of all high example in a ward not less than in a home. Those who learn, as the great and good, the heroic Emperor learnt, and will teach so long as this century is remembered, to suffer silently, are an inspiration and purification to others, who, in admiring, forget themselves insomuch that special consideration creates no jealousy. As to gratitude, were it not for its grace to the giver, it is only too full and constant; yet it is pleasant to be called "sunshine," and to be thanked because one's fairer lot makes one cheery in countenance and of gentle speech. Truly, the reward is great for the weekly expenditure of time needed to amuse or advise the inmates of our too-little visited hospitals. And be the cherished flowers remembered! for, as Tennyson says—

"Flowers to these spirits in prison are all they can know of the spring,
They freshen and sweeten the wards like the waft of an angels wing.
* * * * *
They that can wander at will, where the works of the Lord are revealed,
Little guess what joy can be got from a crocus out of the field."

C. S. D.

### Lectures.

On Friday, July 20th, at the Midwives' Institute and Trained Nurses' Club, a lecture will be delivered at a quarter to eight on "The preparation for, and after-treatment of operations in private." Admission for non-members 6*d*.

### Vacancies.

The following vacancies are announced:—

**Matrons.**

Lying-in Hospital, Liverpool; £50
Gordon Hospital for Fistula.

**Nurses.**

Newport Infirmary; two.

Doncaster Union; £25.

Holborn Union; £20

Fulham Union; £16.

Infectious Hospital, Newcastle; £25

Swansea Nursing Institution.

Sheffield Nurses' Home; several.

Kingston Union. Head Nurse; £40

Blackheath Nursing Institution. Several; £20.

Cornwall Trained Nurses' Home, Truro; two.

Shoreditch Infirmary; £20

Kingston Union; £40.

Ipswich Nurses' Home; £22.

Jersey Institution.

Southport Trained Nurses' Home.

Brompton Cancer Hospital; £25.

Stoke-on-Trent Nurses' Institution; several.

Nursing Institute, St. Heliers, Jersey

Midwives' Institute.

Bristol Royal Infirmary; £40.

**Night Superintendent.**

Bristol Royal Infirmary; £40.

**District Nurses.**

Leeds.
Stockport.

**Probationer.**

Haywood Hospital, Burslem

THE NURSING RECORD.

THE "NURSING RECORD."

A Journal for Nurses and a Chronicle of Hospital and Institution News, &c.

PRICE TWOPENCE. EVERY THURSDAY.

In the interest of a most important profession which is daily increasing in numbers and popularity, it has been found necessary, in order to secure the full and proper representation of the views and claims of those associated with nursing work, to establish the "Nursing Record," a journal which shall in every way faithfully and unreservedly express the opinions and maintain the views of the class it is intended to support.

The Special Objects of the "Nursing Record" are: 1. To deal with matters relating to the nursing profession, more especially those concerning its advancement and consolidation; (2) to protect the general interest of nurses, and to promote their well-being and improvement; (3) to introduce opinion and criticism upon all subjects associated with nursing work; (4) to offer active assistance in the promotion and support of any movement calculated to be of benefit to the nursing profession.

Support and recommendation is earnestly solicited to enable these views to be thoroughly sustained.

SOME PRESS OPINIONS.

"The 'Nursing Record' is a new venture, the first weekly number of which appeared on April 5th. It is described as a journal for nurses, and a chronicle of hospital and institution news. It is, of course, an outcome of the rapidly increasing interest in such works as it relates to, and it seems likely to prove a useful addition to the literature of its class."—*Queen.*

Subscription post free, if paid in advance: Yearly, 8s.; half-yearly, 4s. 6d.; quarterly, 2s. 6d. Specimen copy post free for two stamps.

London: SAMPSON LOW, MARSTON, SEARLE, and RIVINGTON, Limited, St. Dunstan's House, Fetter Lane, Fleet Street, E.C.

**Price Twopence.** **Every Thursday.**

A JOURNAL FOR NURSES AND A CHRONICLE OF HOSPITAL AND INSTITUTION NEWS, &c.

